# A Multifunctional AIE Nanoprobe as a Drug Delivery Bioimaging and Cancer Treatment System

**DOI:** 10.3389/fbioe.2021.766470

**Published:** 2021-11-08

**Authors:** Keqi Hu, Daquan Zhou, Linlin Rao, Peng Wang, Chunxiang Xiang, Feng Chen

**Affiliations:** ^1^ Department of Neurosurgery, Xiangyang Central Hospital, Affiliated Hospital of Hubei University of Arts and Science, Xiangyang, China; ^2^ Department of Pathology, Xiangyang Central Hospital, Affiliated Hospital of Hubei University of Arts and Science, Xiangyang, China

**Keywords:** drug delivery, aggregation-induced emission, imaging, PLA-PEG, T7 peptide, cancer treatment

## Abstract

Of all malignant brain tumors, glioma is the deadliest and most common, with a poor prognosis. Drug therapy is considered as a promising way to stop the progression of disease and even cure tumors. However, the presence of blood brain barrier (BBB) and blood tumor barrier (BTB) limits the delivery of these therapeutic genes. In this work, an intelligent cell imaging and cancer therapy drug delivery system targeting the blood-brain barrier and the highly expressed transferrin receptors (TfR) in gliomas has been successfully constructed, and an amphiphilic polymer (PLA-PEG-T7/TPE) with aggregation-induced emission (AIE) properties has been designed and successfully synthesized. PLA-PEG-T7/TPE self-assembled polymer micelles showed significant AIE effect in aqueous solution with good biocompatibility. Therefore, it can be used for potential biological imaging applications. In addition, drug-carrying micelles showed typical behavior of regulating drug release. Inhibition of cell proliferation *in vitro* showed that the drug-loaded micelles had dose-dependent cytotoxicity to LN229 cells. In the *in vivo* anti-tumor experiment, PLA-PEG-T7/TPE/TMZ had the best therapeutic effect. These results indicated that T7 functionalized PLA-PEG was a promising platform for nasopharyngeal cancer drug combination therapy.

## Introduction

Glioblastoma (GBM) is the most common primary brain tumor of the central nervous system with a high mortality rate, with a 5-year relative survival rate of 4.5% after diagnosis in the United States (Stupp et al., 2017; Ladomersky et al., 2019). Chemotherapy is one of the most commonly used methods to treat glioma (Teresa et al., 2018; Hanna et al., 2020). Unfortunately, many chemotherapeutic drugs often cause serious side effects because they do not distinguish between cancer cells and healthy cells. To overcome this problem, nanoscale drug delivery systems have been developed to enhance the anticancer activity of these chemotherapeutic drugs and reduce side effects (Nuk et al., 2020; Griffith et al., 2021; Malekjani and Jafari, 2021).

At present, most drug delivery systems can only deliver drugs to cancer cells, and the distribution and release of drug delivery systems in cancer cells cannot be monitored. In order to further develop and optimize drug delivery systems, a multifunctional nanomedicine platform for imaging diagnosis and therapy is needed (Zhao et al., 2017; Wang et al., 2020). The usual approach is to integrate fluorescent dyes into the drug delivery system. However, most conventional fluorescent dyes are toxic, and due to their aggregation-induced quenching (ACQ) effect, the fluorescence quenching in the aggregation state greatly limits their applications in biomedical applications (Gang et al., 2009; Su et al., 2020). Therefore, it is necessary to develop new fluorescent molecules to avoid ACQ effect. Recently, Tang and others have developed a new class of fluorophores with aggregation-induced emission (AIE) properties. Unlike ACQ-influenced conventional fluorophores, AIE-based fluorophores, such as tetrastyrene (TPE), exhibit high emission efficiency in the polymerized state (Long et al., 2014; Gao and Tang, 2017; Lin et al., 2017). Using these unique AIE characteristics, many fluorescent probes with AIE characteristics have been extensively studied by Liu et al. (Gao et al., 2017; Mao et al., 2020; Shi et al., 2020; Liu et al., 2021). These probes have strong fluorescence properties and excellent cell imaging capabilities. Therefore, the development of fluorescence delivery systems based on AIE dyes is considered to be a promising strategy for simultaneous imaging and therapy.

Another obstacle is the concentration of chemotherapies in gliomas, which means that drug delivery systems must cross different biological barriers, particularly the blood brain barrier (BBB) and blood tumor barrier (BTB) of gliomas (Fortin, 2012). It has been reported that brain capillary endothelial cells and many malignant tumor cells have overexpression of transferrin receptor (TfR), which is significantly higher than that of other normal cells (Yoon et al., 2017; Arumov et al., 2020; Wan et al., 2020). Since high concentrations of endogenous transferrin may be a competitive inhibition of the transferrin modified gene delivery system (Lee et al., 2010), the peptide T7 targeting the TfR (His-Ala-Ile-Tyr-Pro-Arg-His) was found to have a similar affinity with transferrin (Han et al., 2010; Kuang et al., 2013; Tang et al., 2019). Moreover, T7 peptide is a small molecule of peptide. It has the advantages of easy chemical synthesis, good stability, and little steric hindrance, and it has good clinical application potential. Therefore, we selected T7 peptides to design gene delivery systems that cross the blood-brain barrier and target brain tumors.

In recent years, polymer micelles have been widely used in the field of medicine as a new delivery carrier due to their unique advantages (Liu et al., 2020; Wu et al., 2021). PLA was the most common biodegradable polymer because it was easy to be degraded and produces non-toxic degradation products which can be eliminated by metabolism (Wang et al., 2018; Shang et al., 2019).

In this study, a covalent bond of polyethylene glycol (PEG) was formed on the surface of PLA to modify T7, and PLA-PEG-T7 was obtained for loading temozolomide and tetrastyrene, as well as for cell imaging and drug delivery. The material can be self-assembled into micelles, in which the tetrastyrene core serves as the optical recognition code for cell imaging, and the biocompatible PEG serves as the micelle shell, with long blood circulation time. The tumor targeting effect of PLAA-PEG-T7 as a drug carrier was evaluated *in vitro* and *in vivo*. The intracellular transport of PLA-PEG-T7/TPE/TMZ NPs was systematically evaluated.

## Experimental Section

### Materials

All chemical reagents were purchased from commercial suppliers and can be used without further purification. PLA-PEG-NH_2_ was purchased from Xi’an Ruixi Biotechnology Co., Ltd. Temozolomide and tetraphenylvinyl were purchased from Shanghai Aladdin Biochemical Technology Co., Ltd. (Shanghai, China). In addition, 1-ethyl-3 -(3-dimethylaminopropyl) carbodiimide hydrochloride and N-hydroxysuccinimide (EDC and NHS) were purchased from Macklin Biochemical Technology Co., Ltd. (Shanghai, China). Cell culture reagents included DMEM medium, fetal bovine serum, trypsin, penicillin-streptomycin, and phosphate buffer saline, available from Gibco BRL (Carlsbad, California, United States). Cell Counting Kit-8 (CCK-8) was purchased from Beyotime Institute of Biotechnology (Shanghai, China). Annexin V-PE Apoptosis Kit was purchased from Beckton, Dickinson and Inc. (United States). T7 peptide was synthesized by Sangon Bioengineering Co., Ltd. (Shanghai, China). LN229 (glioma cell line) was provided by Southern Medical University (Guangzhou, China).

### Synthesis of PLA-PEG-T7

The coupling of PLA-PEG-NH_2_ and T7 was achieved by amide reaction. Specifically, 8 mg of EDC and 10 mg of NHS were added to 3 ml of T7 solution and stirred for 4 h. Then, 20 mg of PLA-PEG-NH_2_ was added dropwise to the above solution, and reacted at room temperature overnight. Finally, the co-polymer was precipitated with cold methanol for 12 h and washed out by same solvent and then lyophilized again to obtain pure PLA-PEG-T7.

### Synthesis of PLA-PEG-T7/TMZ/TPE

PLA-PEG-T7/TMZ/TPE nanoparticles were prepared by double emulsion solvent evaporation method. First, 4 mg of TMZ was dissolved in HCl solution (200 μl, 0.1 M) to form aqueous phase. Then 10 mg of PLA-PEG-T7 and 3 mg of TPE were dissolved in 2 ml of dichloromethane (DCM) to form oil phase. The aqueous phase was added to the oil phase and the resulting mixture was emulsified with an ultrasonic processor for 120 s to form the initial emulsion. The emulsion was then dropped into 1% PVA (10 ml) solution and emulsified again. The homogeneous emulsion was stirred overnight at room temperature to devolatilize the DCM. The PLA-PEG-T7/TMZ/TPE nanoparticles were centrifuged at 13,000 × g for 20 min at 4°C and placed in water for subsequent use.

The loading amount of TMZ was characterized by measuring the UV-Vis absorption peak at 327 nm. The linear regression equation (y = 0.0486x + 0.0493, *R* ^2^ = 0.998) of TMZ was obtained through the standard curve.

### Characterization

Chemical structure of PLA-PEG-NH_2_ and PLA-PEG-T7 was characterized by ^1^H NMR spectroscopy (300 MHz, Varian, United States) using Deuterated chloroform (CDCl_3_) as the solvent. The shape, size, and morphology of the synthesized nanomaterials were studied with transmission electron microscopy (JEOL TEM-1210) at 120 kV and Zetasizer Nano ZS (Malvern) apparatus. Fourier transform infrared spectrum of all samples were collected in a PerkinElmer Spectrum 100 FT-IR spectrometer (PerkinElmer Inc., United States) under the transmittance mode with KBr plates. Excitation and emission spectra of samples were recorded using a FLS920P Edinburgh Analytical Instrument. Confocal optical micrographs were analyzed by performing confocal laser scanning microscope (CLSM, Leica SP8, Nikon, Japan).

### *In Vitro* Drug Release

Dialysis bag method was used to study the release of TMZ *in vitro*. Firstly, 1 ml of 6 mg/ml of PLA-PEG-T7/TMZ was placed in a dialysis bag (molecular weight cut off = 2000 Da). Then, the dialysis bag was placed in a 15 ml centrifuge tube with 3 ml PBS (pH = 6.5) as the release medium. The centrifuge tube was placed at 37°C and shaken at 100 rpm to simulate the release of TMZ *in vitro*. Then, 1 ml of the release solution was removed at regular intervals and 1 ml of fresh PBS was added to the centrifuge tube. The absorption value of the liquid at 329 nm, the concentration at the corresponding time point, and the cumulative release rate were calculated.

### Cytotoxicity of PLA-PEG-T7

The cytotoxicity of PLA-PEG-T7 was measured on L929 cells using the CCK-8 assay. Briefly, the L929 cells (5 × 10^3^ cells per well) were seeded in 96-well plates (Corning, United States) for 12 h. The cells were then incubated with fresh cell medium containing PLA-PEG-T7 with concentrations ranging from 0 to 320 μg/ml and incubated for 24 h. After treatment, cells were washed with PBS and added with fresh cell medium containing 10% CCK-8 to all wells. Finally, the absorbance at 450 nm was tested by a microplate reader (Thermo Scientific, United States). L929 cells incubated with RPMI 1640 medium were used as control groups.

### T7 Targeting Ability Assay

Cellular uptake of PLA-PEG-T7/TPE complex with and without T7 functionalization was analyzed to confirm the targeting ability of T7. In detail, LN229 cells were seeded in 24-well plate with a density of 5 × 10^4^ cells/well and incubated in a 37°C humidified incubator (5% CO_2_) for 12 h. Then, PLA-PEG/TPE or PLA-PEG-T7/TPE complex was added to treat the cells and incubated for different time. After each interval (0.5, 1, 2, 4, and 6 h), cells in each group were washed by PBS, trypsinized, centrifuged, and resuspended in 200 μl PBS. Finally, samples were measured using flow cytometry and the corresponding fluorescent intensity was quantified by Flow Jo 7.6.1 software.

### *In Vitro* BBB-Transport Efficiency

In order to test the *trans*-BBB efficiency of PLA-PEG-T7/TPE, an *in vitro* BBB model was established in a transwell cell culture system. The bEnd.3 cells (5 × 10^4^ per insert) were seeded in the upper transwell and cultured for 7 days with a trans endothelial resistance (TEER) exceeded 200 ω·cm (Stupp et al., 2017). Finally, the following channels were detected by confocal microscope under the same optical conditions at different time points. For transport analysis, transfer the cross-well insert to a new 24-well plate containing 5 × 10^5^ LN229 cells. PLA-PEG/TPE and PLA-PEG-T7/TPE nanoparticles were added to the transwell insert and left for 24 h. Then stain with phalloidin staining solution for 1 h. Observation of the co-stained fluorescence signal of TPE and phalloidin with confocal laser scanning microscope.

### Endocytosis Pathway Analysis

For this study, LN229 cell culture was performed as described above. Referring to the method of selecting the dose of each inhibitor, the cells were precultured in DMEM containing 10 μg/ml chlorpromazine, 200 μM genistein, 200 nM wortmannin, or 5 μg/ml cytochalasin B. After 3 h of pretreatment, the culture medium was taken out, the PLA-PEG-T7/TPE complex containing inhibitor was added, and the culture was continued for 2 h. Cells treated with PBS or incubated at 4°C were set as controls. Next, the cells are trypsinized and centrifuged for flow cytometry detection. FlowJo software was used to analyze the average fluorescence of each group compared with untreated cells.

### Inhibition Cell Proliferation *in Vitro*

Temozolomide (TMZ) was a commonly used anti-tumor drug, which has a strong toxic effect on cancer cells. In order to measure the effect of co-administration of PLA-PEG/TMZ/TPE on LN229 cells survival rate, CCK-8 assay was performed. Briefly, LN229 cells were planted into 96-well plate at a density of 1 × 10^4^/cell. Next, 12 h after seeding, the cells were exposed to PLA-PEG/TMZ/TPE with drug concentrations for 24 h. Then, 100 µl of CCK-8 solution (10%) was added to each well and incubated at 37°C in 5% CO_2_ atmosphere for 1 h. Next, absorbance of each sample was measured at 450 nm using a microplate reader.

### Apoptosis Assay

LN229 cells were inoculated in 24-well plates with a density of 5 × 10^4^ cells/well and cultured overnight in an incubator. The original medium was then replaced by complete medium containing PBS, TMZ, PLA-PEG-T7, and PLA-PEG-T7/TMZ/TPE and incubated for 24 h. After the experiment, the cells were washed with PBS and digested with trypsin, then the supernatant was removed by centrifugation, and the cells were resuspended in 200 μl binding buffer and stained for 15 min by adding 5 μL Annexin V-PE and 5 μl 7-AAD. The apoptosis of the cells in different treatment groups was analyzed by flow cytometry.

### Cell Cycle Assay

In short, LN229 cells were cultured as described in the cell uptake test. Then, the cells were treated with various preparations (PBS, PLA-PEG-T7, TMZ, PLA-PEG-T7/TMZ). After 24 h, the cells were fixed with 70% ethanol. Before analysis, the fixed cells were stained with ribonuclease A and propidium iodide. Next, we analyzed the cells by flow cytometry and analyzed the cell cycle by cell cycle analysis software.

### Scratch Healing Assay

LN229 cells were seeded in a 24-well plate at a density of 5 × 10^4^ cells/well and cultured overnight. After culturing overnight, a line was created on the bottom using a 10 μL pipette tip. PLA-PEG-T7, PLA-PEG/TMZ, PLA-PEG-T7/TMZ suspended in serum-free DMEM were used to culture LN229 cells. Serum-free DMEM was used as control group. The scratch was photographed for different time intervals (0, 12, and 24 h) using an inverted fluorescence microscope.

### Biocompatibility Evaluation

#### Morphology of Red Blood Cells

Firstly, fresh whole blood was centrifuged at 1,000×g for 5 min, and the lower layer of red blood cells (RBC) were collected. Three concentrations of PLA-PEG-T7 (0.1, 0.2, and 0.5 mg/ml) were reconfigured by PBS, and PBS served as negative control group. Each sample was mixed with an appropriate amount of red blood cells and incubated for 1 h. The red blood cells were collected by centrifugation, washed with PBS, and then fixed by 4% paraformaldehyde for 1 h.

As for the SEM sample preparation, fixed erythrocytes were placed on glass slides and dehydrated with 70, 85, 95, and 100% ethanol in turn. The dehydration time was 10 min each time. After the treated samples were naturally air-dried, then sprayed with gold, and the morphologies of red blood cells were observed by scanning electron microscopy (SEM).

#### Hemolysis Assay *in Vitro*

PBS aqueous solution of PLA-PEG-T7 with different concentrations of 0.1, 0.2, and 0.5 mg/ml was prepared. Deionized water and PBS were used as positive and negative controls, respectively. Next, 4 ml of samples with different concentrations were put into a centrifuge tube, and 200 μl of 16% red blood cell suspension was added for co-incubation. The supernatants were collected by centrifugation at the preset time points (1, 3, 5, 8, 18, and 24 h) at 1,000×g for 5 min. The absorbance of each sample at 540 nm was measured by microplate analyzer, and the hemolysis rate was calculated by the following formula:

Hemolysis rate (%) = (A-C)/(B-C) ×100%

where A, B, and C represented the absorbance of the experimental group, the positive control group, and the negative control group respectively (three groups were parallel for each sample).

#### APTT and PT

In total, 270 μl of platelet lean plasma was mixed with 30 μl of PLA-PEG-T7 solutions of different concentrations (0.01, 0.1, 0.2, and 0.5 mg/ml in PBS) at room temperature, and the corresponding detection reagents were added. The activated partial thromboplastin time (APTT) and prothrombin time (PT) of each mixture were determined by automatic coagulation analysis instrument.

### *In Vivo* Fluorescence Imaging

The animal experiment was carried out with the help of Jinan University and all animal experimental protocols have been approved by the Animal Protection and Use Committee of Jinan University. For evaluating the targeting ability of PLA-PEG-T7, tumor-bearing nude mice with a tumor size of about 100 mm^3^ were selected and injected 100 μl of PLA-PEG-Cy5 complex and PLA-PEG-T7-Cy5 complex via tail vein. At different time intervals, the nude mice were subjected to fluorescence imaging using the small animal live imager (FX Pro Bruker). At 8 h after injection, the nude mice were sacrificed to take out hearts, livers, spleen, lungs, kidneys, and tumors, and then the main organs and tumor were imaged (excitation wavelength: 630 nm, emission wavelength: 700 nm) *ex vivo*.

### Tumor Inhibition Assay

For the tumor suppression test, tumor-bearing mice were randomly divided into five treatment groups: PBS control group, PLA-PEG-T7, TMZ, PLA-PEG/TPE/TMZ, and PLA-PEG-T7/TPE/TMZ. Each group included three mice that were injected every 2 days. Tumor volume was measured with an electronic caliper and calculated as 1/2 × shortest diameter (Stupp et al., 2017) × longest diameter. At the same time, the body weight of the mice was recorded. After 10 treatments, the tumors were collected and weighed.

### Histological Analysis

The collected tumors were immersed in tissue fixator for routine paraffin embedding and cut into tissue sections with a thickness of 4 μm. Then, the sections were stained with hematoxylin and eosin (H&E). For immunohistochemical analysis, the level of tumor apoptosis was evaluated by the terminal deoxynucleotidyl transferase deoxyuridine triphosphate nick end labeling (TUNEL) assay. In addition, the expression of Ki67 and CD31 in tumor sections were also detected.

#### *In Vivo* Biocompatibility Evaluation

Histological analysis: After the mice were euthanized, the heart, liver, spleen, lung, kidney, and other major organs were collected, immersed in paraformaldehyde fixative, embedded in conventional paraffin, and sliced into 4 μm thick tissue sections. Then the sections were stained with hematoxylin and eosin and imaged with a fluorescence microscope.

Blood chemistry assay: After 21 days of treatment, the mouse blood was collected and centrifuged at 3,000 rpm for 5 min to obtain the serum in the supernatant. Then, the kidneys (UREA, UA, and CREA), liver functions (AST, ALT, and ALB), blood glucose (GLU), Low Density Lipoprotein Cholesterol (LDL), and myocardial enzymes (LDH) were evaluated through blood parameters.

## Results and Discussion

### Synthesis and Characterization of Products

Transferrin receptor (TfR) is highly expressed in brain capillaries. Targeting TfR has long represented an attractive way to penetrate the BBB (Ullman et al., 2020). Both PLA and PEG are FDA-approved biodegradable polymer materials that can be used in the human body and have good biocompatibility. In this study, PLA-PEG micelle was designed and grafted T7 peptide to target transferrin receptors at brain capillaries to penetrate BBB.

**FIGURE 1:**
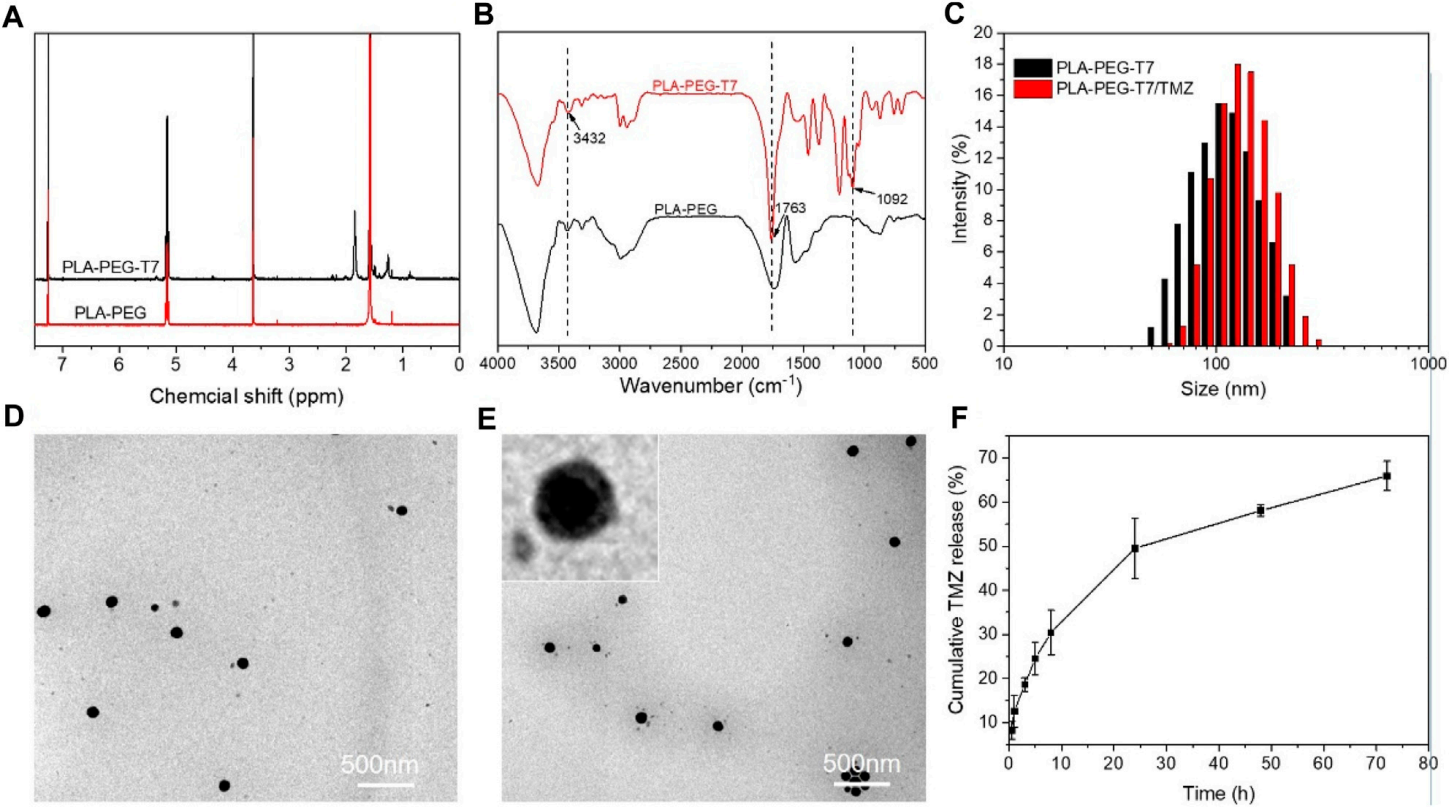
Characterizations of PLA-PEG-T7. **(A)** ^1^H NMR spectra of PLA-PEG and PLA-PEG-T7. **(B)** FTIR spectra of PLA-PEG and PLA-PEG-T7. **(C)** Size distribution of PLA-PEG-T7 and PLA-PEG-T7/MZ. **(D)**TEM image of PLA-PEG-T7. **(E)** TEM image of PLA-PEG-T7/TMZ. **(F)** Cumulative TMZ release (%) from PLA-PEG-T7/TMZ in PBS buffer (pH = 6.5) values at different time intervals.

T7 was grafted on PLA-PEG micellar through amide reaction. Figure 1A showed the ^1^H NMR spectra of PLA-PEG and PLA-PEG-T7. The chemical shift that occurred at 7.3 ppm corresponded to the solvent peak of CDCl_3_. Chemical shift at 5.1–5.2 ppm corresponds to the H atom on the last methyl of the lactic acid unit, 3.6–3.7 ppm corresponds to the H atom on the CH_2_ of the block copolymer polyethyleneglycol unit, 1.5–1.6 ppm corresponds to the H atom on the block methyl of polylactic acid unit, and 1.9–1.7 ppm and 1.3–1.2 ppm newly appeared peaks are a series of proton peaks on T7^32^. FTIR measurement showed that PLA-PEG-T7 was successfully synthesized (Figure 1B). The absorption peak at 1,092 cm^−1^ was attributed to C-O-C stretch of PEG segments. The strong absorption peak at 1756 cm^−1^ belonged to -C=O stretch, indicating the formation of PLA segments. The broad absorption peak at 3,243 cm^−1^ was -OH stretching, which was practically eliminated from the spectrum of PLA-PEG block copolymers. The results showed that T7 was successfully grafted onto the block polymer.

DLS and TEM image showed that PLA-PEG-T7 micellar had average diameter of about 100 nm and the distribution was uniform (Figures 1C,D). After loading with TMZ, the micelles still remain regular spherical shape. DLS showed that the particle size of PLA-PEG-T7/TMZ increased slightly and TEM image showed a diameter distribution of about 100–200 nm (Figures 1C–E). PLA-PEG-T7 and PLA-PEG-T7/TPE/TMZ were suspended in PBS to observe the stability. And the change in average particle size was measured by DLS within 24 h. As shown in Supplementary Figure S1, the diameter of the PLA-PEG-T7/TPE/TMZ micelles changed little from 106 to 135 nm, behaved higher stability than PLA-PEG-T7 alone.

### Drug Release

Due to the layered structure of PLA-PEG-T7, hydrophobic drugs TMZ could be encapsulated in the cavity structure of PLA-PEG-T7 during the formation process, and the loading amount of TMZ in the PLA-PEG-T7/TMZ composite was 69 mg/g. In order to investigate the release behavior of PLA-PEG-T7/TMZ, acidic environment (pH = 6.5) was prepared to stimulate the tumor tissue. TMZ was slowly released in the first few hours, but increased after 8 h. By 72 h, the cumulative drug release reached 65%, indicating that PLA-PEG-T7 could release the drug slowly and solve the problem of sudden drug release (Figure 1F). Previous reports using PLA-PEG micelle to carry Paclitaxel (PTX), the release curve is similar to this study. Nearly 85% PTX released to PBS (pH = 5.0), while 70% PTX released to PBS (pH = 7.4) (Cai et al., 2020). The higher release amounts owing to the more acidic environment.

### *In Vitro* BBB-Transport Efficiency

**FIGURE 2:**
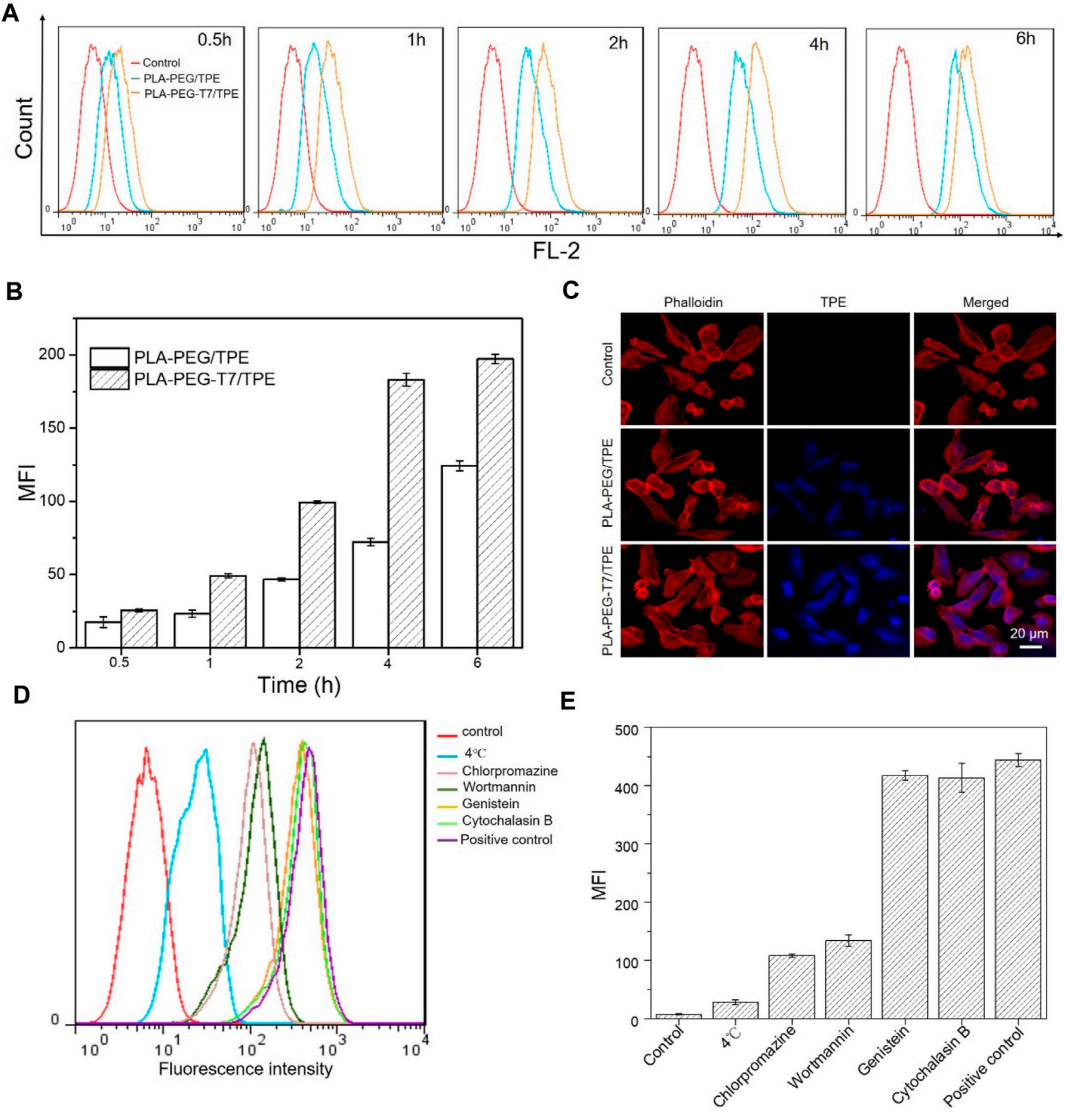
**(A)** Flow cytometry analysis of fluorescence peak figure in LN229 cells incubated with PLA-PEG/TPE and PLA-PEG-T7/TPE for different time. **(B)** Mean fluorescence intensity (MFI) in LN229 cells incubated with PLA-PEG/TPE and PLA-PEG-T7/TPE for different time. **(C)** Confocal laser scanning microscopy (CLSM) shows the uptake of TPE into LN229 cells between PLA-PEG/TPE and PLA-PEG-T7/TPE a groups in a two-compartment BBB model. **(D)** Flow cytometry analysis of the effect of various inhibitors on the endocytosis of PLA-PEG-T7/TPE into LN229 cells. **(E)** The effect of various inhibitors on the endocytosis of PLA-PEG-T7/TPE in LN229 cells.

The unique BBB of the brain makes it difficult for traditional chemotherapy to achieve better therapeutic effects. It is urgent for drugs to effectively penetrate the BBB to target tumor cells and exert therapeutic effects. As a tumor-targeting peptide, T7 peptide can bind to TfR with significantly greater affinity (Tang et al., 2019; Yu et al., 2019). The *in vitro* BBB-crossing efficiency of PLA-PEG-T7/TPE in the transwell system was shown in Figure 2C. CLSM showed that compared with the PLA-PEG/TPE group, the PLA-PEG-T7/TPE group had more TPE accumulation in the cytoplasm, which indicated that PLA-PEG-T7/TPE nanoparticles crossed the BBB more easily.

### T7 Targeting Ability Assay

Efficient uptake of by *in vivo* cells was the key to achieving the biological performance of material design (Patalag et al., 2021). To verify the targeting of T7 on LN229 cells, cell uptakes of PLA-PEG/TPE and PLA-PEG-T7/TPE were also compared by flow cytometry as shown in Figure 2A. As expected, the fluorescence intensity of PLA-PEG-T7/TPE with T7 function was strongest at the same time point, indicating a significant increase in the absorption of PLA-PEG-T7/TPE by LN229 cells (Figure 2B). This suggests that T7-targeted peptides can significantly enhance the endocytosis efficiency of the PLA-PEG-T7/TPE nano micelle.

### Endocytosis Pathway Analysis

For nanocarriers, endocytosis is a prerequisite for entering cells, and endocytosis is usually achieved by phagocytosis, macrocytosis, clathrin-dependent endocytosis, and clathrin-independent endocytosis. In order to explore the pathway of the PLA-PEG-T7/TPE complex into LN229 cells, several specific drug inhibitors were used, such as chlorpromazine (an inhibitor of clathrin-dependent endocytosis), genistein Flavonoids (inhibitors of vesicle-mediated endocytosis), wortmannin (specific inhibitors of macrocytosis), and cytochalasin B (specific inhibitors of phagocytosis) to block specific endocytosis pathways. The results of the endocytosis pathway were shown in Figures 2D,E. Similar to other reports, low temperature (4°C) significantly inhibits cellular uptake compared with the normal 37°C, indicating that the endocytosis process is energy-dependent. In addition, chlorpromazine also significantly inhibits the uptake of PLA-PEG-T7/TPE by LN229 cells, and the fluorescence intensity was reduced by 70% compared with the control group, indicating that clathrin-dependent endocytosis is the main endocytic pathway for PLA-PEG-T7/TPE complex entering LN229 cells.

### Cytotoxicity of PLA-PEG-T7

**FIGURE 3:**
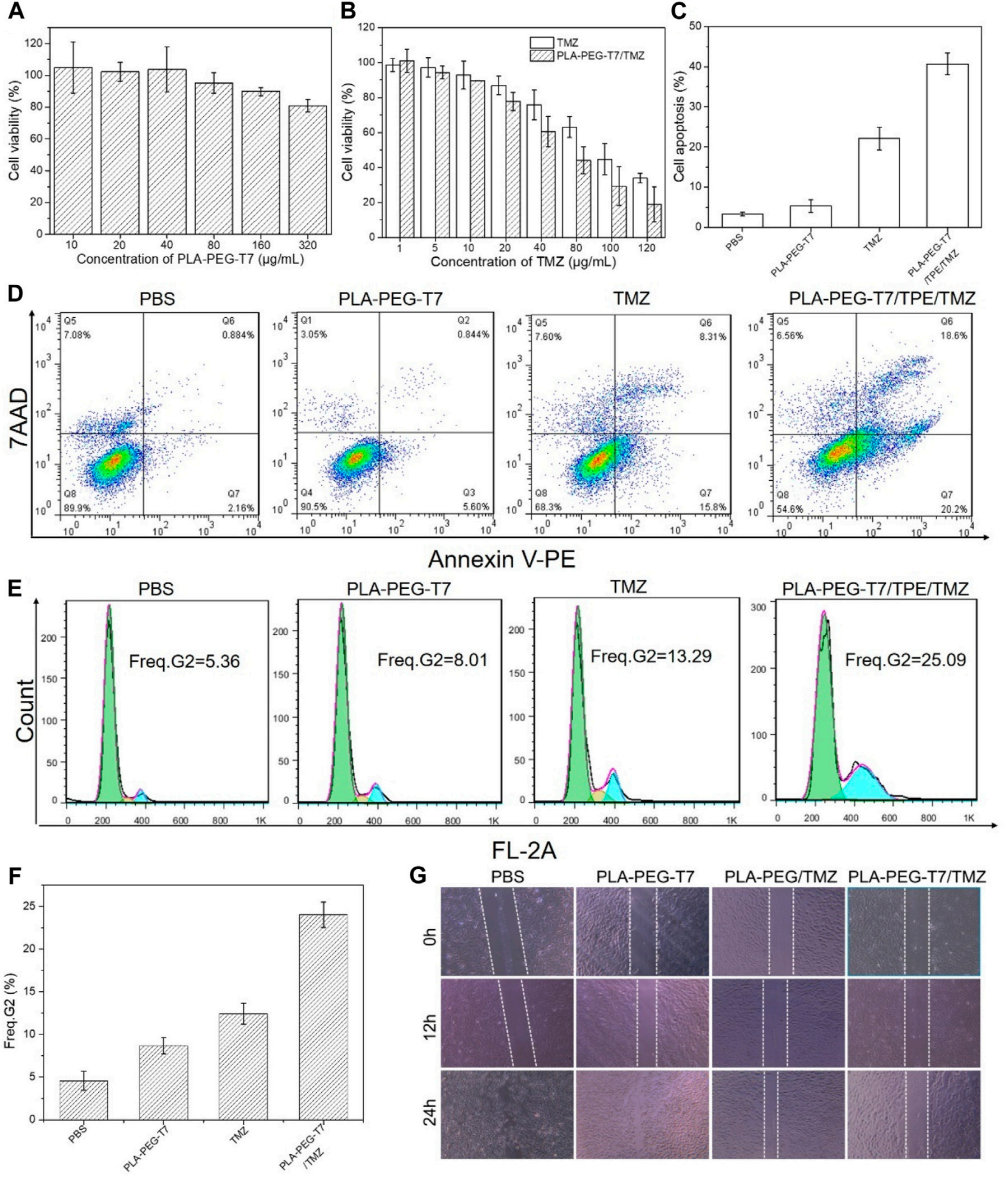
**(A)** *In vitro* cytotoxicity of PLA-PEG-T7 with different concentrations on LN229 cells over an evaluation period of 24 h. **(B)** Cell viability of LN229 cells pretreated by TMZ and PLA-PEG-T7/TMZ with different concentrations. **(C)** Result histogram of apoptotic LN229 cells. **(D)** Apoptosis analysis by flow cytometry after LN229 cells incubated with various formulations using Annexin-V PE and 7-ADD dual-staining method. **(E)** Flow cytometry analysis of cell cycle in LN229 cells. **(F)** Freq. G2 of cell cycle in LN229 cells. **(G)** Scratch diagram of LN229 cells after treatment in different experimental groups.

For *in vivo* applications, an important requirement for nanocarriers was to have low cytotoxicity. In order to investigate the cytotoxicity of PLA-PEG-T7 *in vitro*, CCK-8 method was used in this study to detect the survival rate of L929 cells under different concentrations of PLA-PEG-T7. When the concentration was 80 μg/ml, the survival rate of cells was 95%, indicating the toxicity of copolymer to cells was very small. With the further increase of polymer concentration, cell viability gradually decreased. Even at high concentrations (320 μg/ml), the survival rate of cells was more than 80%, which means that the PLA-PEG-T7 nano micelle had good biocompatibility (Figure 3A).

### Inhibition of Cell Proliferation

The CCK-8 method further confirmed the feasibility of PLA-PEG-T7/TMZ to inhibit the proliferation of LN229 cells *in vitro*. After 24 h incubation, the *in vitro* cytotoxicity of TMZ and PLA-PEG-T7/TMZ was determined. As shown in Figure 3B, different experimental groups had inhibitory effects on cells and were concentration-dependent. The results showed that TMZ and PLA-PEG-T7/TMZ had anti-tumor activity against LN229 cell lines at appropriate concentrations. Compared with TMZ alone, PLA-PEG-T7/TMZ showed better tumor inhibition effect.

### *In Vitro* Apoptosis Assessments

We also used flow cytometry to study the apoptotic effect of PLA-PEG-T7/TMZ on LN229 cells. LN229 cells treated with different preparations were stained with Annexin Ⅴ-PE and 7-ADD. Flow cytometry quantitatively analyzes the apoptotic cell population after drug treatment. As shown in Figures 3C,D, after 24 h of incubation with blank PLA-PEG-T7, the apoptosis of these cells was negligible compared with the PBS control, indicating that it was non-toxic to LN229 cells at the measured concentration. TMZ and PLA-PEG-T7/TMZ could significantly induce LN229 cell apoptosis. The apoptotic rates of TMZ and PLA-PEG-T7/TMZ cells were 20.13% and 42.46%, respectively. Compared with TMZ, the apoptosis rate of LN229 cells treated with PLA-PEG-T7/TMZ was significantly increased. These results indicate that the nanocarrier loading strategy can effectively deliver drugs to cells and induce tumor cell apoptosis, which has potential application prospects in the comprehensive treatment of tumors.

### Cell Cycle

The DNA content was analyzed by flow cytometry to determine the effect of PLA-PEG-T7/TMZ on cell cycle progression. As reflected in the G2 cell population shown in Figures 3E,F, no significant difference was observed in the cells treated between the blank PLA-PEG-T7 and the PBS control. However, the cells treated with TMZ showed a significantly larger cell population in the G2, 13.9% or 25.09%, respectively, which was much higher than the 5.36% of the PBS control, indicating that TMZ can inhibit intracellular DNA replication and induce cell apoptosis.

### Scratch Healing Assay

Through the wound healing test, the inhibitory effect of PLA-PEG-T7/TMZ on the migration of LN229 cells was studied. As shown in Figure 3G, after 24 h of incubation, complete wound closure of blank PLA-PEG-T7 was observed. However, the cells treated with TMZ still retained a large area of uncovered wound. For the drug delivery system, the largest uncovered wound area was observed, indicating that PLA-PEG-T7/TMZ can effectively inhibit the migration of LN229 cells and help improve the anti-tumor efficacy.

### *In Vivo* Fluorescence Imaging

**FIGURE 4:**
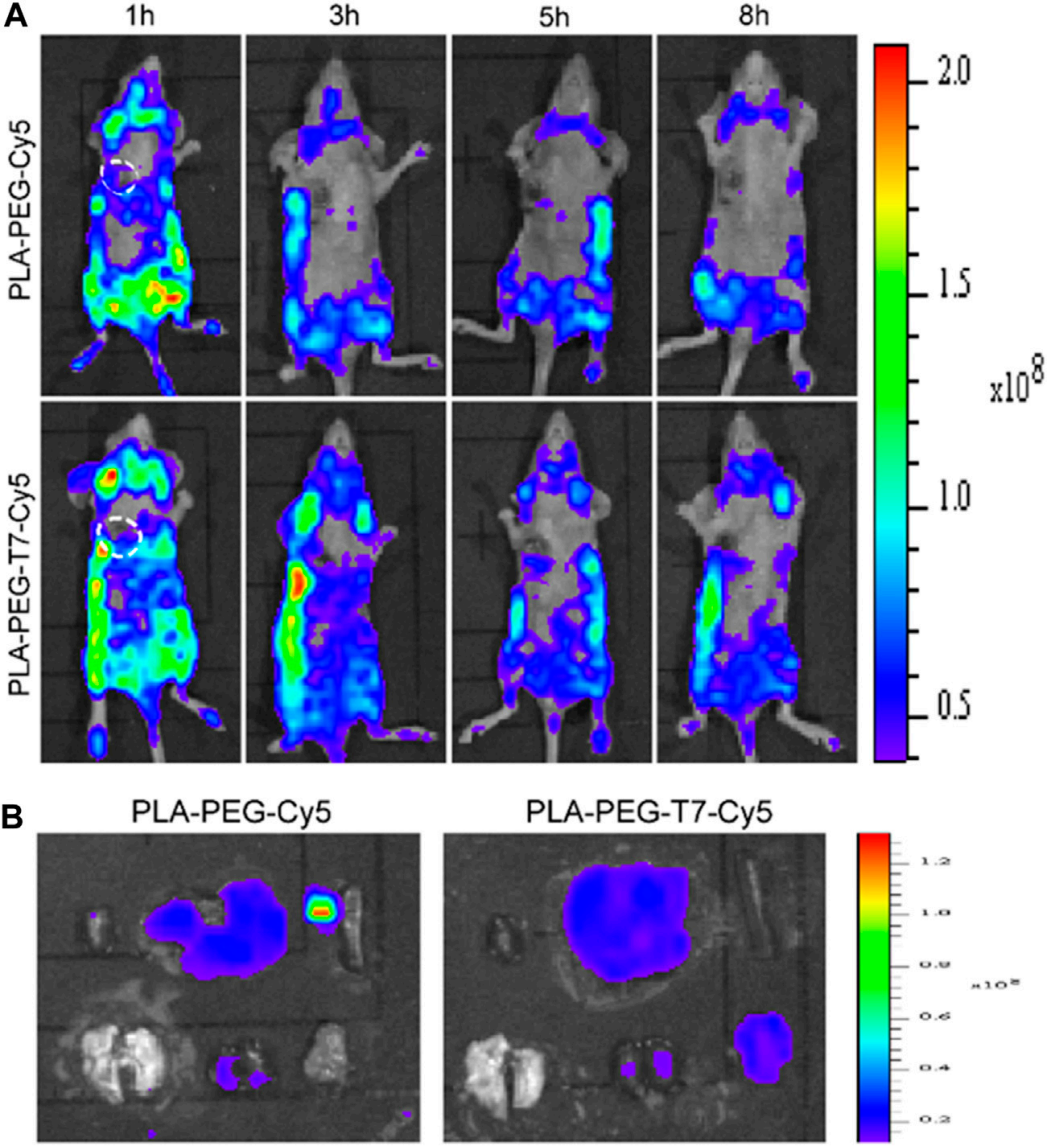
**(A)** *In vivo* fluorescence images of LN229 tumor-bearing nude mice at different time points. **(B)** *Ex vivo* fluorescence imaging of various organs and tumors of LN229 tumor-bearing mice after injection.

The targeting ability of PLA-PEG-T7 was evaluated by fluorescence imaging experiments. The experimental results are shown in Figure 4A. Compared with PLA-PEG-Cy5 without targeting, PLA-PEG-T7-Cy5 modified with T7 was injected intravenously for 1 h, when the tumor site has obvious fluorescent signal. The fluorescent signal at the tumor site was significantly enhanced 3 h after the injection. The experimental results showed that the modification of T7 enhanced the material’s tumor targeting. At 8 h after injection, the nude mice were sacrificed, and tumors and normal tissues were collected for *ex vivo* imaging (Figure 4B). The tumors treated with PLA-PEG-T7-Cy5 showed obvious fluorescent signals, indicating that the nanomaterials were delivered to the tumor.

### *In Vivo* Assays

**FIGURE 5:**
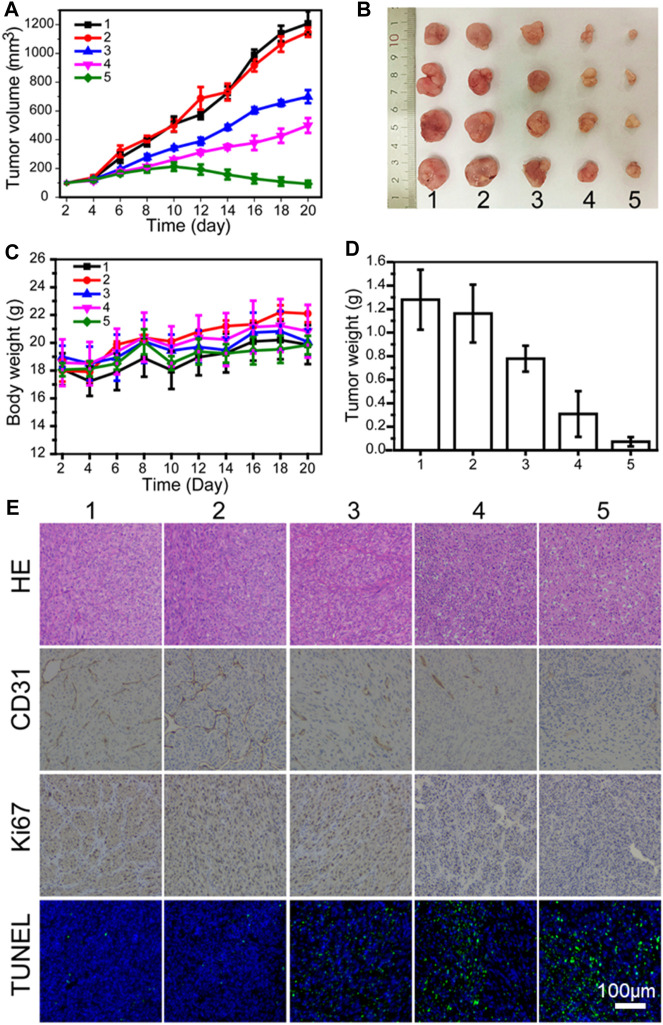
**(A)** *In vivo* tumor growth curves of LN229 tumor-bearing mice treated with different formulations. **(B)** Representative image of LN229 tumors at the 21st day. **(C)** Body weight changes of mice treated with different formulations during the treatment. **(D)** The tumor weights excised from different groups after 21 days treatment. **(E)** Immunohistochemical analyses of H&E, TUNEL, CD31, and Ki67 for LN229 tumor tissues after the last treatment with different formulations *in vivo* (200 ×) (1: PBS; 2: PLA-PEG-T7; 3: TMZ; 4: PLA-PEG/TMZ/TPE; 5: PLA-PEG-T7/TMZ/TPE).

Encouraged by the good combination therapy of LN229 cells outside the recipient, the anti-tumor effect of PLA-PEG-T7/TPE/TMZ was studied through the treatment of nude mice carrying LN229 tumors. Figures 5A,B show representative tumor images and tumor growth curves treated with various preparations. It was found that the blank PLA-PEG-T7 had no effect on the growth of the LN229 tumor, and there was no significant difference in the growth curve of the PBS control. The group treated with TMZ showed significant anti-tumor effects. In particular, tumors treated with PLA-PEG-T7/TPE/TMZ showed a smaller volume than tumors treated with free TMZ, indicating that PLA-PEG-T7/TPE/TMZ drugs can enter LN229 cells more due to the targeting effect, and showed better anti-tumor effect *in vivo*, which is consistent with the *in vitro* apoptosis test. That means PLA-PEG-T7 delivery of drugs may be a promising strategy for the treatment of cancer. During the *in vivo* experiment, the changes in the weight of the mice were recorded, and it was considered to be a key factor reflecting the safety and side effects of the single formula food used. As shown in Figure 5C, the weight of each group of mice increased, so the PLA-PEG-T7/TPE/TMZ preparation was safe for mice during *in vivo* tumor treatment.

### Histologic and Immunohistochemical Analysis

The hematoxylin-eosin (H&E) staining method was also used to analyze the tumor necrosis after the material treatment. H&E images showed that PLA-PEG-T7/TPE/TMZ treatment induced the largest necrotic tumor area, in which the largest number of nuclei were deformed (Figure 5E). A terminal deoxynucleotidyl transferase-mediated dUTP nick end labeling (TUNEL) assay was performed to further evaluate the *in vivo* apoptotic effects of different treatment methods. Similarly, PLA-PEG-T7/TPE/TMZ induced the highest percentage of apoptosis-positive tumor cells, confirming its strongest anti-cancer activity *in vivo*. CD31 and Ki67 immunohistochemical staining were also performed on each group of tumor sections to monitor the changes in blood vessel density and tumor tissue proliferation activity, respectively. The CD31 stained images showed that the tumor tissue vascular density was greatly reduced after PLA-PEG-T7/TPE/TMZ treatment, indicating that APLA-PEG-T7/TPE/TMZ can effectively inhibit the angiogenesis of tumor tissues. The Ki67 stained image indicated that PLA-PEG-T7/TPE/TMZ treatment also significantly inhibited the proliferation activity of tumor cells.

**FIGURE 6:**
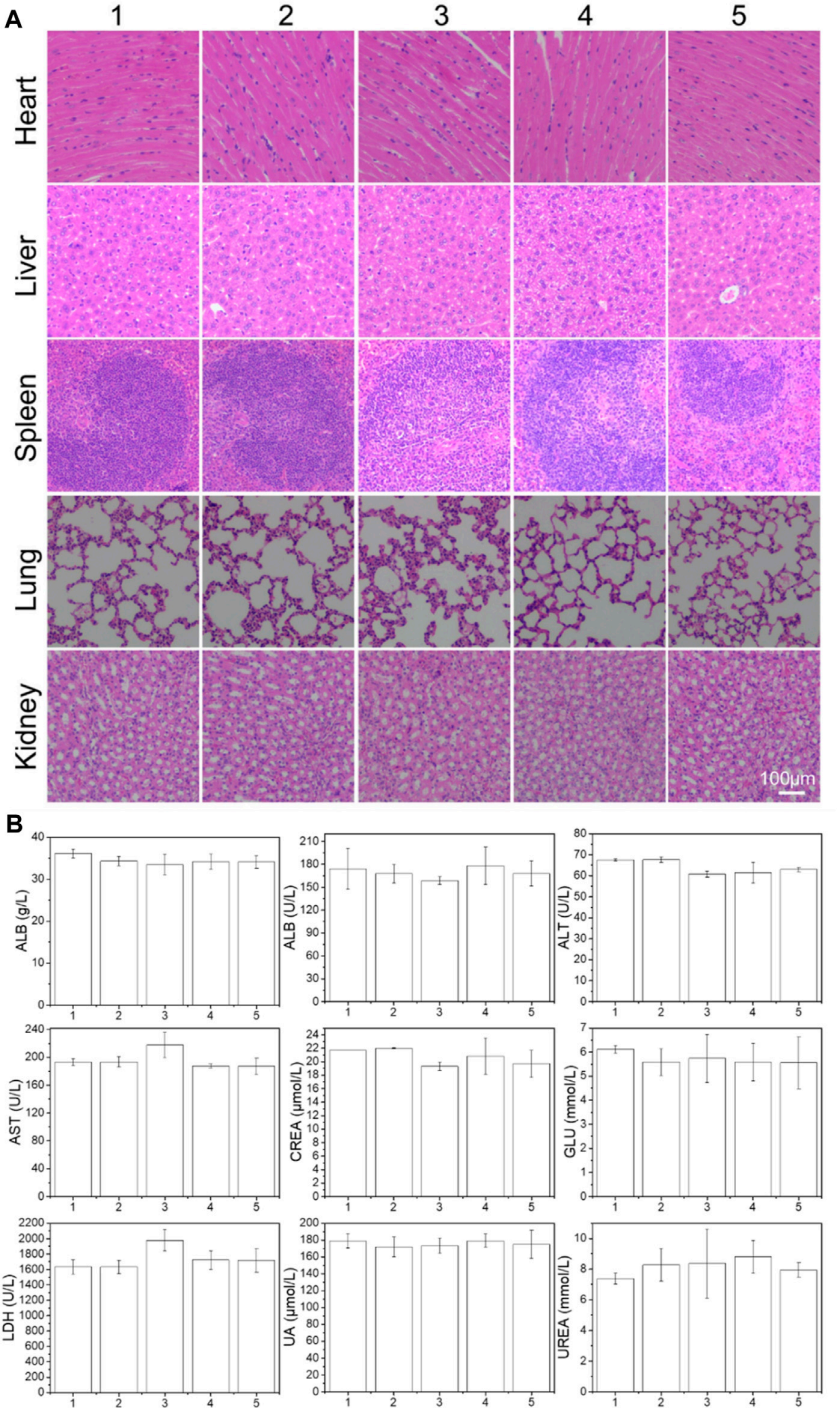
**(A)** Histologic assessments of major organs in mice (200×) treated with different formulations. **(B)** Serum levels of albumin (ALB), alkaline phosphatase (ALP), alanine aminotransferase (ALT), aspartate aminotransferase (AST), lactate dehydrogenase (LDH), glucose (GLU), creatinine (CREA), uric acid (UA), and urea (UREA) of mice after 21 days treatment of different formulations. (1: PBS; 2: PLA-PEG-T7; 3: TMZ; 4: PLA-PEG/TMZ/TPE; 5: PLA-PEG-T7/TMZ/TPE).

In addition, major organs were excised from BALB/C nude mice for further histological analysis to evaluate the safety of the preparation. A representative photograph of the H&E stained sample was shown in Figure 6A. Compared with the PBS control, most of the preparations treated organs have no visible damage, indicating that most of the preparations have good biological safety. This result once again confirmed the safety and necessity of the PLA-PEG-T7 vector.

The blood chemistry parameters used to assess kidney, liver, blood lipids, blood sugar, and myocardial enzyme functions in each group were also tested (Figure 6B). Compared with PBS control mice, no significant changes in blood parameters were observed in the mice treated with PLA-PEG/TMZ/TPE and PLA-PEG-T7/TMZ/TPE. Therefore, we concluded that the PLA-PEG-T7/TMZ/TPE complex is a safe material and is expected to be used in the diagnosis and treatment of cancer *in vivo*.

### Biocompatibility

**FIGURE 7:**
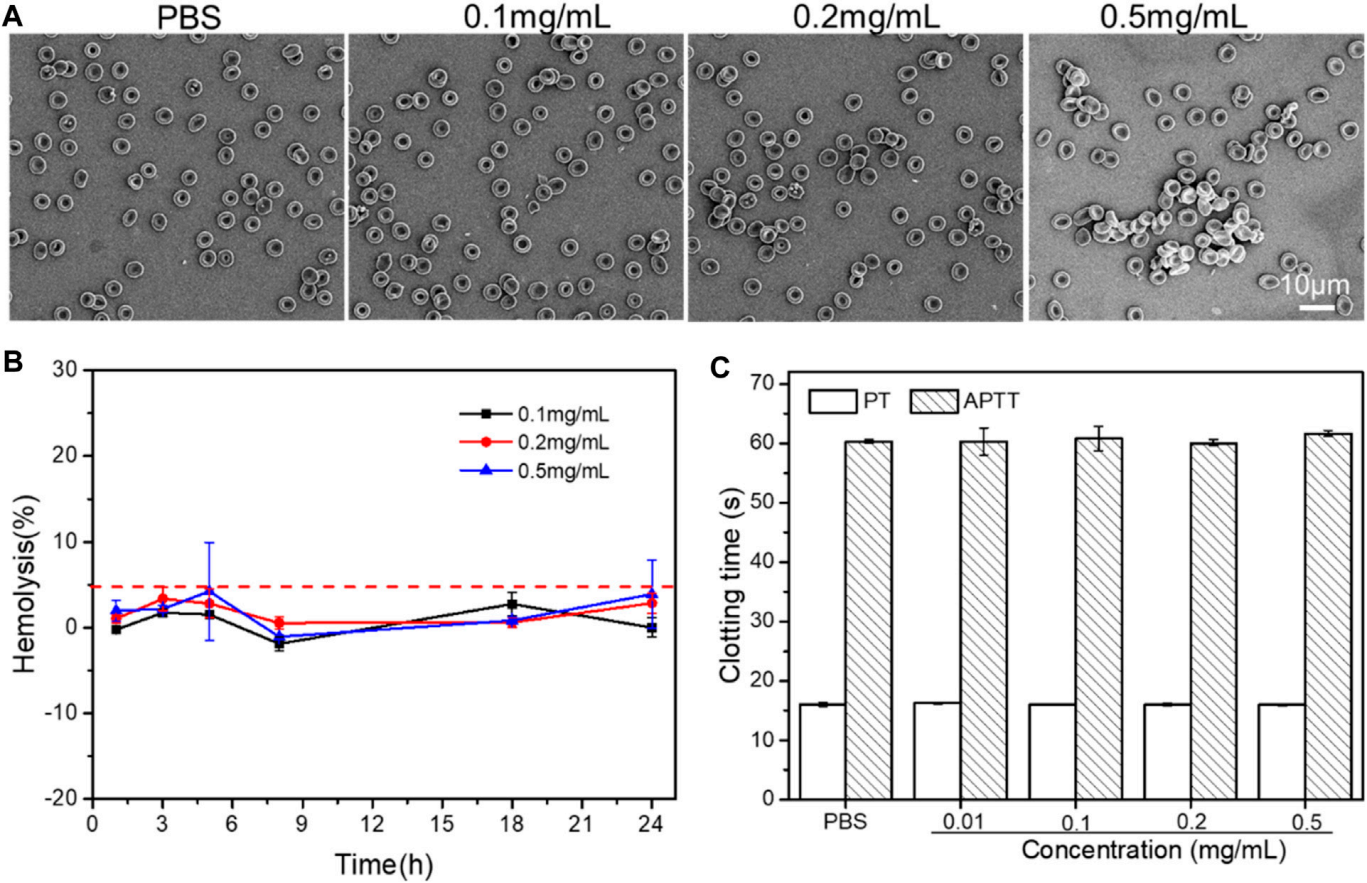
**(A)** Effect of PLA-PEG-T7 with different concentrations on the aggregation and morphology of RBCs. **(B)** Effect of PLA-PEG-T7with different concentrations on the hemolysis. **(C)** Effect of PLA-PEG-T7with different concentrations on the APTT/PT.

Through hemolysis test and red blood cell morphological changes, the blood compatibility of PLA-PEG-T7 was studied. As shown in Figure 7B, 0.5 mg/ml PPLA-PEG-T7 did not produce any hemolysis (the hemolysis rate was less than 5%). In addition, the effect of PLA-PEG-T7 on red blood cells was further evaluated. As shown in Figure 7A, the excellent blood biocompatibility of PLA-PEG-T7 is further confirmed. The results showed that in the concentration range of 0.1–0.5 mg/ml, the morphology of red blood cells did not change after treatment with PLA-PEG-T7. The reason why PLA-PEG-T7 has excellent blood biocompatibility is the presence of PEG fragments.

In addition, APTT/PT analysis was performed to further evaluate the blood safety of PLA-PEG-T7. As shown in Figure 7C, compared with the PBS group, different concentrations of PLA-PEG-T7 had no obvious effect on APTT and PT. It proved that PLA-PEG-T7 had good blood compatibility within a specific concentration range.

## Conclusion

Polylactic acid-polyethylene glycol bioabsorbable micelles have many advantages as a drug delivery system, such as biocompatibility and sustained release of drugs. This research developed a new micellar carrier for co-delivery of TMZ and TPE. Drug-free micelles do not affect the viability of cells. For the anti-tumor effect of PLA-PEG-T7/TPE/TMZ, it was found that PLA-PEG-T7/TPE/TMZ was much better than the free TMZ formulation used. PLA-PEG-T7/TPE/TMZ has a good inhibitory effect on the proliferation of LN229 cells *in vitro*, and a good inhibitory effect on LN229 tumors *in vivo*. In addition, PLA-PEG-T7 shows non-toxicity and excellent blood compatibility, indicating that it is a promising drug delivery vehicle in the comprehensive treatment of tumors.

## Data Availability

The raw data supporting the conclusions of this article will be made available by the authors, without undue reservation.
