# Supplementary material for: A Multifunctional AIE Nanoprobe as a Drug Delivery Bioimaging and Cancer Treatment System
Source: Front Bioeng Biotechnol. 2021 Nov 8;9:766470. doi: 10.3389/fbioe.2021.766470 (PMC8606747; doi:10.3389/fbioe.2021.766470)
Supplement: Supplementary file 1 [file DataSheet1.docx]

Figure S1. The stability of PLA-PEG-T7 and PLA-PEG-T7/TPE/TMZ in PBS.

PLA-PEG-T7 and PLA-PEG-T7/TPE/TMZ were suspended in PBS to observe the stability. And the change in average particle size was measured by DLS within 24 h. The diameter of the PLA-PEG-T7/TPE/TMZ micelles changed little from 106 nm to 135 nm, behaved higher stability than PLA-PEG-T7 alone.
